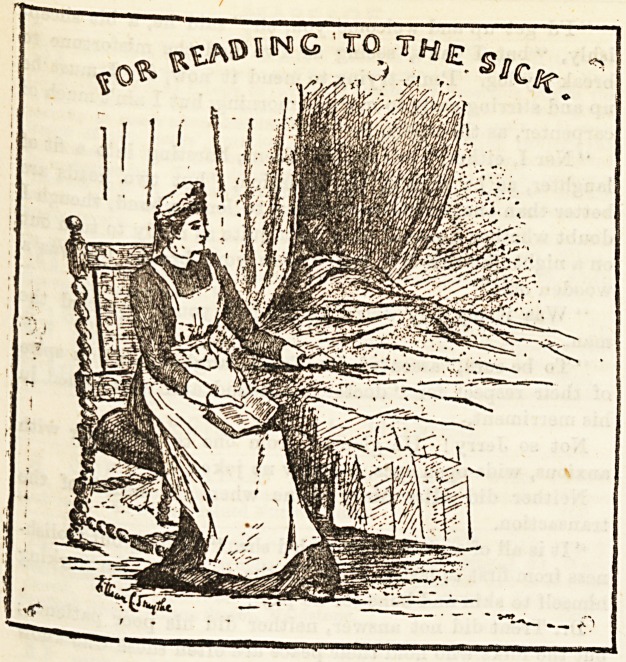# The Hospital Nursing Supplement

**Published:** 1891-08-01

**Authors:** 


### "The Hospital" Nursing Mirror.

Being the Extra Nursing Supplement of "The Hospital" Newspaper.

Contributions for this Supplement should be addressed to the Editor, The Hospital, 140, Strand, London, W.C., and should have the word "Nursing" plainly written in left-hand top corner of the envelope.

### En Passant.

TRAINED NURSES' CLUB.—A large number of nurses availed themselves of the cordial invitation given by this Club to any members of the second thousand who needed a haven of rest during their sojourn in London last week. Both the pleasant Club rooms in Buckingham Street were freely opened, and greatly did the nurses appreciate the advantages offered. Cloaks and bonnets were left, tea was drank, and many restful hours and much pleasant conversation was enjoyed.

SHORT ITEMS.—We have been obliged to hold over many matters this week owing to the pressure on our space.—Three bouquets were presented to the Princess of Wales on Saturday, and two have been acknowledged, but Miss Knollys has mislaid the name and address of the presenter of the third bouquet; therefore will any nurse who having given a bouquet has not had it acknowledged, please send her name and address to Miss Knollys?—A conference of workers amongst women will be held at Birmingham in November; amongst the Presidents are Mrs. S. G. Rathbone and Mrs. Booth.

TO PENSION FUND NURSES.—On Saturday last several members of the Second Thousand expressed, what they declared to be a general wish, to get up another screen for presentation; we therefore make the following suggestion: That a screen of photographs be presented to H.R.H. the Princess of Wales, the President of the Fund, providing that 1,000 members of the First and Second Thousand each send their photograph (carte) and a postal order for 2s. to "Screen," Miss Pritchard, The Lodge, Porchester Square, London, W., by August 28th. The names will be received in the order in which they are sent; nurses are requested to write their names and their policy number on the back of the photographs. With a view to saving expense all photos and sums will be acknowledged in these pages, and not privately. If the 1,000 photos are not forthcoming, the money will be returned.

JEWISH NURSES.—In 1889 we had several notes on the need of nurses in the Jewish community, and the subject was ventilated in various papers, but, alas! it seems that the man with the will to carry out suggestions was wanting, and the subject fainted and died away. Now, again, it is raised in the pages of the *Jewish Chronicle*, and let us hope that this time someone will take hold of the scheme and not let it end in talk. The editor of the above paper points out that at the Jewish Home for Incurables the whole of the nursing has to be done by Gentiles, owing to the absolute lack of Jewish experts. The fault must be entirely with the Jewish women of the middle class, for it is well known that there are none more interested in nursing than Lord and Lady Rothschild, Mr. F. D. Mocatta, and other supporters of our great hospitals and institutions; and the doors of the London Hospital are open for the training of Jewish probationers, the kitchen in connection with the Hebrew wards there removing all difficulties with regard to food. There is absolutely no reason for the lack of Jewish nurses, unless some scruple amongst the women themselves, which we Christians cannot comprehend, holds them back from the labour the life of a nurse entails. Such a scruple, however, cannot be a worthy one. Communications on the subject are requested by F., care of Mrs. Morris Joseph, 9, Canfield Gardens, Finchley Road, N.W.

THE TRAINING OF NURSES IN MEDICAL ELECTRICITY AND MASSAGE.—Particulars of the summer term examination at Grafton College, Fitzroy Square, have reached us, and we note that one of the pupils, an M.D., obtains high honours in electricity and electro-therapeutics, and another passes in the second division with credit. The practical examination seems to have been useful, for each student was given a patient and a prescription, and requested to first ask for the apparatus and accessories he or she thought necessary for the proper performance of the treatment prescribed. These being supplied, the treatment had to be carried out under the eye of two of the examiners. Nurses who pass this examination must know how to carry out in an intelligent manner the instructions of medical men regarding treatment by electricity and massage.

ANOTHER PICNIC.—On Tuesday, July 14th, the nurses and visitors staying at the Brassey Holiday Home had a most enjoyable picnic; the place chosen for it was Ecclesbourne Valley. They started from the Home at eleven o'clock in two carriages for those who were unable to walk both ways. The valley was reached shortly after twelve o'clock, when the hampers, which were laden with all good things, were quickly unpacked, and the company seated themselves on the grass, and partook of a hearty meal, which was much enjoyed. After luncheon they separated into different parties and rambled to see the different scenes belonging to that delightful spot. At four o'clock they all returned and found tea waiting for them, which was highly appreciated. After tea the party all went down for a ramble on the beach, and enjoyed it very much, and at half-past six the carriages came and they returned home.

THE NURSES' CO-OPERATION.—A long article on "Trained Nurses," by Miss Clementina Black, has appeared in some provincial papers. The writer says, amongst other things: "The quality of food provided varies, I am inclined to think, a good deal. Much, in this matter, depends upon the individual steward or Matron. Peculating stewards and careless Matrons have existed, and will exist again, and nurses and patrons alike will suffer through their failings. Such nurses, however, as I have known personally, have found the food good and sufficient in the hospitals to which they belonged, and I think this may be taken to be the case on the whole with all large hospitals that are freely visited by the public." Further on, Miss Black speaks of the co-operation of Nurses in favourable terms, and her opinion on this subject is well worth having: "I am glad to say that the Nurses' Co-operation has flourished greatly in the few months of its existence. Early in May there were 103 nurses on its books, and every one of them was out. A pile of telegrams asking for nurses stood on the Lady Superintendent's desk when I called to see her, and another arrived during our short interview. The Co-operation has also male attendants, and masseurs and masseuses on its books." In the *Queen* the following notice of the Co-operation appeared: "The Nurses' Co-operation at 8, New Cavendish Street, has supplied nurses for over 400 cases in the last three months. The Treasurer's report to the General Committee was exceedingly satisfactory, and the Co-operation may claim to have proved that the large percentage exacted from their nurses by the institutions is exorbitant and unjust."

### The Second Thousand.

### At Merchant Taylors' Hall.

There was some witchery abroad in the City in the dusk of Thursday evening; the members of the Stock Exchange had gone west to their dinners, the Bank was closed and its clerks had vanished, and Black Maria had long since carried off its last load from the Mansion House. The terrific rush of the City day was over, but the usual stillness did not fall on that open space, for from all sides there came streams of figures in nursing uniform, and the soft sound of women's laughter arose on the still air, causing a belated clerk's hair to rise on end with astonishment. This stream of women invaded Merchant Taylors' Hall, and flowing into the great central room there, began to assume some sort of order. Six hundred of the Second Thousand members of the Royal National Pension Fund were being directed as to the order into which they were to approach their President, the Princess of Wales, to receive their certificates from her hands. General Crease and four of the sergeants of the Royal Marines and two of the Royal Artillery with great good nature instructed the nurses in the simple duties of forming fours and detachments of 100, and answering promptly to their numbers. This little bit of work was enlivened by the strains of the City of London Police band, and as soon as it was got through the Countess Cadogan entered on Mr. Burdett's arm and took her place on the platform at the end of the hall. Other visitors grouped behind her included Lord Cathcart, Dr. Bristowe, Dr. Steele, and Mr. Bryant. All the nurses then marched up, were presented to Lady Cadogan, and from her received their card of invitation to Marlborough House for Saturday. This was a very picturesque scene in the handsome hall, and evidently attracted the attention of two or three artists present, the sketches of the representative of the *Daily Graphic* appearing on Saturday last.

When all the nurses had passed, Mr. Burdett proposed a vote of thanks to Lady Cadogan for attending and presenting the invitations, and, in doing so, spoke of the enrolment of a second thousand nurses as a "great fact" in reference to the future, and expressed a hope that the pending reception by the Princess of Wales would help all the nurses present to realise and act up to their responsibility. Dr. Bristowe (St. Thomas's Hospital) then made a short impromptu speech, stating that the Fund had succeeded marvellously, though without Mr. Burdett's aid it must have been a failure. It had been opposed in many ways, and by people who ought to have known better. One Matron refused to ask her nurses to join because she said "The Almighty would look after the weak." Dr. Bristowe, in conclusion, congratulated the nurses on the ceremony in which they were going to take part on Saturday. Shortly after Lady Cadogan (who is one of the Vice-Presidents of the Fund) took her departure.

A number of visitors began to arrive, and in one of the rooms a concert was going on; a harp and piano duet by Miss Dixon and Miss Jenkins, a quartette of male voices, and a song by Mrs. Dick being received with much applause. There was a jingle of coffee cups in the different refreshment rooms, and general welcoming and meeting of old friends, and then a sudden rush to the central hall once more to hear Mr. Harry Furniss in his "Humours of Parliament." This proved to be a most amusing entertainment, and many of the nurses sat for two hours watching the illustrations and listening to the descriptions of how our legislators behave. At least, how they behave as seen by the eyes of Mr. Harry Furniss, for we confess that in the House itself we never saw one tithe of the "humours" which the lucky lecturer has been privileged to perceive. Meanwhile the Police Band had moved into the quadrangle, another concert was taking place upstairs, and the coffee cups had been replaced by wine glasses and sandwiches. The refreshments were under the charge of Messrs. Ring and Brymer, and were really admirably managed. A crowd of tired and hungry nurses would swoop down on a table and clear all before them. But in a moment fresh piles of strawberries and jugs of champagne-cup appeared, and all was ready for the next onslaught. Passing and repassing were many faces well known in the nursing world; Mr. Clifford, wherever he moved, was besieged by questioning nurses; Miss Sprigg, in evening dress, wandered about in search of members of her institute in New Bond Street; Mrs. Suckling, from Winchester; Miss Heanley, from Boston; Miss Cross, from the Evelina; Miss Durham, with her medals; Mrs. Dacre Craven; Mrs. Henrietta Walker, in her pretty grey uniform; Miss Culverhouse, Miss Raynor, Mr. Almond Hind, Mr. Michelli, Dr. Saville, and many others. It was certainly a most enjoyable evening. But there is one sad confession to be made in conclusion, and that is that the cloak-room was quite inadequate for the rush made on it by some six hundred women, and something very like a scramble and catch-who-catch-can happened with the bonnets and cloaks. We sincerely hope no great damage was done, and that in the end each nurse managed to secure her own belongings. The fact is, women are, as a rule, a minority in the City, and the elaborate arrangements were made for taking the gentleman's coats; and lo! there stood empty their room, while the ladies' room, up a narrow stair, was besieged, and in the end blockaded. The thanks of all are due, and we are sure are heartily given, to the Merchant Taylors' Company for the loan of their splendid suite of rooms, to all who helped in the concert-room, to General Crease and his sergeants, to the railway companies for reduced fares, and to the stewards, many of whom worked hard at the hall to make the evening as enjoyable as possible to the nurses. As to the patience and the courtesy displayed by all the officials of the fund, it was untiring, and the result was seen on Saturday, when the whole ceremony passed off in a simply splendid manner.

### At Marlborough House.

Saturday dawned a lovely day—regular Queen's weather—and all dread of an indoor ceremony and limp, rain-bedraggled uniforms vanished immediately. Towards noon 'busses and cabs took the nurses to Marlborough House, and an inquisitive crowd gathered at the garden entrance to watch the nurses passing in. Miss L. C. Smythe and Mr. Pocock were early on the scene, and, with the help of Mr. Bryant, arranged the certificates in a huge stack at one side of the conservatory entrance. The nurses formed into companies, under the same sergeants who had been present on Thursday night, and a few visitors began to arrive. Invitations had been issued to the Vice-Presidents of the Fund, to the heads of the College of Physicians and the College of Surgeons, to thirty Matrons of metropolitan hospitals, and to a few others connected with nursing. So that there gathered under the trees opposite the house a small group, which included Mrs. Dacre Craven, Miss Stewart (of St. Bartholomew's), Miss Phillippa Hicks (of the Nurses' Co-operation), Mrs. and Miss Burdett, Mrs. G. Q. Roberts, Miss Monk, Miss Cook, Miss Gibson, Miss Busby, Miss Walker (of the London), Miss Evans (of Berrywood Asylum), Dr. Broadbent, Dr. Bristowe, Mr. Bryant, Mr. Murray Ind, Dr. Steele, and others.

Punctually at half-past twelve the Prince and Princess of Wales, accompanied by the Princesses Victoria and Maud of Wales, Prince George of Greece, the Countess Cadogan, Lord Rothschild, and with Lady Suffield, Miss Knollys, Lord Colville of Culross, General Sir Dighton Probyn, and Colonel Clarke in attendance, came out of the conservatory. The Princess took her place on the lowest step, and the first detachment of nurses was called forward. They came in alphabetical order, so that the uniforms were mixed; here was Miss Taylor, Matron of the East London Nursing Society, in her black gown and black bonnet and veil, and next a Queen Charlotte's nurse in her pure white uniform. Then the nurses of St. John's House, Worcester, wearing their badges. Then came a pretty brown dress and cap with brown ribbons, her "own fancy" in uniform of a private nurse, and next to her the pretty fluffy caps of the St. John's nurses, and then a group of Gloucester Infirmary nurses. Miss Paget, with her Queen's badge on her arm, and Miss Dunn, of Dublin, with her Queen's badge, and then one of the Army Nursing Sisters, from Shorncliffe, in her pretty red cape; then two nurses in the South Devon uniform. One nurse had come from the Canary Isles, several from Glasgow, and one from Castletown, in the Isle of Man. There were several smiling Winchester nurses, particularly pleased because their generous committee had paid all their expenses; and then in a group came the twelve attendants from Berrywood, in blue with scarlet facings, looking like so many picturesque vivandières. As they came up the Prince turned to Lady Cadogan to ask who they were, and in truth it was impossible not to be struck by their smart uniform, so unlike the quiet greys and browns affected by those who minister to the sick in body, not in mind. Last of all came Sister Frances, of Vancouver, of whose work our readers have heard much lately.

Each nurse having received her certificate from the hands of the Princess, the Prince stepped forward, and bidding the nurses draw near, addressed them as follows:—

Nurses, it has afforded the Princess and myself much pleasure to receive you here to-day, as the representatives of the second thousand nurses who have joined the Royal National Pension Fund. I understand that you come from all parts of the United Kingdom, and that, whereas a large proportion of the first thousand nurses were attached to institutions, by far the larger proportion of the second thousand are working on their own account as private nurses. Our fund is intended not only for nurses working within the United Kingdom, but for all nurses throughout the British Empire. The Colonies are fairly represented amongst the policy-holders who have joined the Fund, and I am informed that there is one nurse here to-day who has come all the way from Vancouver. We are most anxious that nurses working in the Colonies, and that those who employ nurses there, should take an interest in the Royal National Pension Fund, which has the will and the power to render them material assistance. We have to regret the absence of a large number of nurses to-day (though we cannot but rejoice that their sense of duty has prevented them from leaving their cases), owing to the great amount of severe illness with which this country is unfortunately afflicted at the present time. A year ago we were able to welcome the first thousand nurses, and it is not a little remarkable that the second thousand should have placed their savings in the Fund in the twelve months which have passed since we last had the pleasure of receiving you here. I am glad to be able to congratulate you on the steady progress which has been made, and I desire especially to commend to your attention the new features which have been so wisely introduced by the Council. They include the establishment of the Benevolent Fund, which has already £10,000 to its credit and an income of upwards of £400 a year, which will be employed to afford immediate pecuniary or other relief, by loan or absolute gift, to Matrons, Sisters, and nurses, if members of the Pension Fund, who may be in distress, and to assist them in keeping up the payment of premiums on any policies they may have taken out in the Society. Annuities will also be granted to Matrons, Sisters, and nurses who, from no fault of their own, may be or are unable to provide for themselves after sixty years of age. This benevolent Fund has been established as a memorial to your benefactor and friend, the late Mr. Junius S. Morgan, whose memery will be cherished by the nurses as well as by all who are connected with this Society. Formerly, private nurses found it difficult to invest their savings in the Fund, owing to the irregularity of their earnings and their consequent inability to bind themselves to make regular payments month by month. To meet such cases it has been determined to permit nurses to pay irregularly into the Fund such amounts as they may be able to deposit with it. When the sum deposited amounts to £10 it will be applied to the purchase of a paid-up policy in the Fund on the returnable scale, or to the payment of premiums on existing ordinary policies, as each nurse may prefer. Nurses are also permitted to withdraw with interest any funds which they may entrust to the Council. Finally, the policy-holders now have direct representation on the Council, and as the Fund is a mutual one, it is not altogether surprising that it should have proved so popular with the nursing body as a whole. We have now invested funds which amount altogether to £100,000, and there seems every reason to believe that the phenomenal progress so far attained will continue and increase as time goes on. The Princess takes the deepest interest—the deepest *personal* interest (here the Prince glanced at the Princess, who smiled and bowed)—in the success of this most beneficent Society, and we both hope that it may continue to grow and increase until it embraces not only the whole nursing body, but the large proportion of all workers who spend their lives in ministering to the needs and requirements of the sick throughout the British Empire.

Lord Rothschild, one of the Vice-Presidents, said he was proud, on behalf of the Patronesses, Vice-Presidents, and Council of the Fund to return thanks to the Prince and Princess for their gracious reception of the nurses, as it was an honour to be present on that day to testify to the gratitude of the whole nation for the interest which their Royal Highnesses, as well as the other members of the Royal family, took in every institution which was for the good of Her Majesty's subjects. Only a year ago their Royal Highnesses received the first thousand nurses, and it was a remarkable testimony to the popularity of the Fund that within the year one thousand more policies had been taken up by the nurses and others. This satisfactory progress was perhaps in a large measure due to the great personal interest which their Royal Highnesses had taken in the Society as the nurses who were received at Marlborough House last year carried the fame of the Fund into every part of the country, and so proved most successful advocates. The Prince's speech had dealt so exhaustively with the features of the Fund that there was only one point left for him to deal with. Whilst the general public and the nurses had responded most enthusiastically to the scheme for their benefit, the response from the hospital authorities in seeking affiliation with the Fund had been less rapid than could be wished. He desired to convey, more especially to the Princess, whose great personal interest in the Fund had been more useful to its progress than words could express, the thanks of the nurses for the honour conferred upon them by their Royal Highnesses, and the pleasure they had given them that day. The Royal family of England made themselves conspicuous by the whole-hearted devotion which they displayed in developing good works of all kinds, and he was confident there had never been a movement with which they had been associated more conducive to good than the promotion of the success of the Royal National Pension Fund, in which the Prince and Princess of Wales had shown the greatest personal interest from the first.

The Royal party then withdrew, and the nurses proceeded to the marquees, where a cold luncheon was served; the band of the 1st Life Guards softly commenced "Ché faro," chairs were placed beneath the trees, and the garden party aspect was assumed. And how pretty it was to see the neatly clad nurses in their white caps and aprons, passing through sunshine and shadow across the smooth lawns, and grouping on the green banks! These servants of the sick, the honoured guests of our future queen—what a lesson in charity and courtesy to all the world, is taught by the royal regard of true worth which the Princess of Wales has shown! For Her Royal Highness is no careless dispenser of her favours, and no fickle friend to the cause to which she has once lent her support; to win the approbation of the Princess of Wales is to attain absolute security.

Lady Cadogan spoke to several of the nurses, and presently Miss Knollys came out and stated that the Princess was so tired she had been obliged to lie down, and would be unable to appear again. General regret and sympathy was expressed; the nurses pressed forward to beg Miss Knollys to present the Princess with three bouquets they had brought, and to convey to Her Royal Highness their warmest thanks for her kindness, and their sorrow at hearing she was fatigued. Presently the band played "God Save the Queen," and the nurses, gathering under the Princess's window, gave three gentle cheers, waved their handkerchiefs, and departed. The last cab rolled away, and the reception at Marlborough House of the Second Thousand became a subject for conversation, and a pleasant memory to remain with them all for many a long year.

### Cape Colony.

Dr. Greenlees' letter, published on July 18th, has brought us a tremendous rush of correspondence, in answer to which we now give all the information we possess about Cape Colony as a field of labour for English attendants and nurses. First, there is Dr. Duncan Greenlees' assertion that the climate at Grahamstown is excellent, and that the attendants at the Asylum where he is in charge there, are better paid than in England. If any attendants want to go out, they had better write direct to Dr. Greenlees; there is a Secretary for the Cape of Good Hope at 112, Victoria Street, S.W.; or information can be had from the Emigrant's Information Office, 31, Broadway, Westminster. There was also published in 1887 "The Cape of Good Hope General Directory and Guide Book," which might prove useful; but we may as well state at once that it is no use for helpless people to apply for help. If a man or woman is incapable of finding out where to go for information, where to apply for the sailing of ships and the cost of passage—and, in fact, want all their work done and their success assured to them, they had better stay at home and be thankful if they can earn bread and butter. Emigration always means a certain amount of risk, and is only suitable for those strong in mind and body, who can be cheerful through difficulties, and are not likely to suffer from home sickness. We have known many nurses who have gone to Cape Colony; some have failed, some have succeeded. There was the little batch that went out with Miss Mollett to Johannesberg; the home they started proved a failure and went from bad to worse, and was finally closed; some of the nurses are now home again; others, full of the spirit of adventure, have gone on to Mashonaland, while yet others are working soberly at Kimberley. To mention some of the hospitals: we last week recorded the appointment of Miss Hickman to be Matron of the Cottage Hospital, King Williamstown, at a salary of £80. Miss Pagett was appointed Matron of the Nursing Institution, Pretoria, about two years ago; Miss Magee, about the same time, was appointed Matron of the Albany Hospital, Grahamstown. Sister Mary Agatha, three Sisters, twelve nurses, and one probationer, nurse the new Somerset Hospital, at Cape Town. There are 150 beds. Mrs. Vibert is Matron of the Durban Hospital and Mrs. McDonald, of the Grey's Hospital, Pietermaritzburg. Of the Kimberley Hospital, over which Sister Henrietta reigns, we gave a long account in our issue for March 14th; and as for Johannesberg, if any one wants to lead another forlorn hope there, we advise them to read an article published in our issue for November 15th, 1890. One of the most satisfactory letters we ever received from Cape Colony was from a midwife who went out to Port Elizabeth; she held the diploma of the Obstetrical Society and also a certificate from a provincial hospital. She got plenty of work at once at a high rate of pay; she had of course to face various difficulties, to nurse without appliances, to drive long distances over bad roads, to put her hand to cookery, to try and ignore fleas, and to fight against dirt; but she was a cheerful woman with a big mind, and so she smiled and succeeded, and was a favourite everywhere. Nursing Institutions seem to be unknown in Cape Colony; Sister Henrietta sends out private nurses sometimes, but as a rule the private nurse has to trust to her own resources. At Barbeton there is a Private Hospital with a staff of the Matron, two nurses, and two probationers; the Matron receives £120 a-year, the nurses £50 a-year. Miss Mackay, Matron of the Frere Hospital, East London, receives £125 a-year; there are twenty-five beds. Miss Lucy Aisbitt is Matron at Jagersfontein. Now, will nurses and attendants please believe we have told them all we know on the subject of Cape Colony, and if any of them go out will they write and tell us how they succeed? The best equipment for success we are sure is good certificates and testimonials and introductions, good health, and good spirits.

### Everybody's Opinion.

[*Correspondence on all subjects is invited, but we cannot in any way be responsible for the opinions expressed by our correspondents. No communications can be entertained if the name and address of the correspondent is not given, or unless one side of the paper only be written on.*]

### ST. JOHN THE EVANGELIST.

"An Old St. John's Nurse" writes: May I be allowed to say that the Sisters of St. John the Evangelist have four ranks, not two as stated in The Hospital, July 18th. The Grey Sisters—Associates of first and second degree, one wearing a small copper cross, the other a metal cross like your illustration; and the Blue Sisters, Probationer, and Confirmed, the former wearing a cross of silver like the illustration; the latter were the only ones who had the cross with the eagle, and were supposed to belong entirely to the Sisterhood. The nurses wore the narrow ribbon for three years, and the broad one after that time.

### The Princess of Wales' Fund for Mrs. Grimwood.

The following sums are acknowledged:—Members of the Midwives' Institute and Trained Nurses' Club, £1 1s.; H. Cooper, 3s.; Ada Middleton, E. H. Edwards, and Nurse Wilkes, 2s. 6d. each; Nurses A. C. Stewart, Barbara Chapman, M. Wilson, Louisa Webb, Maunder, E. D. Buxton, and One of the First Thousand, 2s. each; Nurses A. Waters, Talbot, F. Kidd, M. Cress, Janie May, Annie Swift, Brierly, Rose, E. R. S., Lucy Wing, C. E. Parkes, Annie Moore, S. Wylie, and Sister Margaret, 1s. each. The list closes next week, so any nurses desiring to contribute are requested to send a postal order for 1s. to "Mrs. Grimwood Fund," Miss Knollys, Marlborough House, London, S.W.

### Presentations.

Miss Crane, late Charge Nurse at the Holborn Workhouse Infirmary at Mitcham, having resigned, after a service of about seven years under the Holborn Board of Guardians, was on the 14th inst. presented with a gold ring as a small token of esteem from her fellow officers, and with every good wish for her future prosperity.

On the occasion of her leaving the Royal Infirmary, Edinburgh, to fill the post of Head Nurse in the English Hospital at Rio Tinto, Nurse Ferrier was presented by the students working in wards 17 and 18, with a valuable case of instruments and a number of useful books, including Hilton's "Rest and Pain," Caird and Cathcart's "Surgical Handbook," Humphry's "Manual of Nursing," &c. The instrument case bore a suitable inscription.

### MEMORY.

It is very difficult when we are ill to remember anything consoling, or even pleasant, to amuse ourselves with, for generally our throbbing heads and aching bones fill all our thoughts. Or else foolish things that we wish we had never said or done, and silly tales and songs, come back and annoy us, while sometimes even ghastly tales we have read and the frightful figures they conjure up before our mind's eye help to make day and night alike wretched.

Now, we have often heard that there is a God who made us, and who, though He may correct us for our good, is always able and willing to help us in our need, but we have thought little about Him in health. Now in sickness, however, we are just finding out what a blessing it would be if we could realize this fact, and we try to recall a hymn, or a little prayer learnt in childhood at our mother's knee. As they rise to our lips we for the first time call on our Father which is in heaven from our very hearts. And this loving Father sees our efforts, and knows that we are longing to return to Him whom we have neglected, perhaps despised, so long. He opens His arms to us, and gladly answers the feeblest petition made to Him in faith. We must encourage every thought which turns to Him, and not let these little beginnings of better things wither away. The memory is such a wonderful engine for good or for evil in our lives that we should be very careful what we put into its keeping, for nothing is ever lost that has once been stored there, but will in the future start up before us for our weal or woe. When, please God, we are restored to health, we will at once set about making a collection of valuables which shall last us to the end of our lives; we may even begin now. What treasure shall we seek first? Our Lord Jesus answers—Seek first the kingdom of God and His righteousness; so we will begin by committing short passages of Holy Scripture to memory, or hymns which express our love, faith in, and reverence for our Saviour and our God; then in our ailments we shall be comforted as we recall that the same Christ who healed the sick, made the deaf to hear, the dumb to speak, and the lame to walk, is still as mighty to save as in the days of old. We may go on to store up the good thoughts of holy men, and the brave deeds of those who have laid down their lives for the truth and for the benefit of their fellow-creatures. We must, on the other hand, be careful to keep out anything that will offend either God or our neighbour, such as proud thoughts, or covetous and greedy ones, idle talk, or worse, which we will not so much as name, for these things are the moth and rust which corrupt. We may go on adding to our treasures till the last day of our lives, and to keep our memories fragrant we will copy the wise housekeeper, who puts camphor to preserve her garments and kill the insects which would otherwise feed on them. God's love to us is this medicine and preservative in one, while our love to man is the sweet lavender which perfumes all around. With such a store to fall back upon we can never be at a loss in sickness or in health, but, like the scribe, will be able to bring out of our treasury things new and old.

### Appointments.

[It is requested that successful candidates will send a copy of their applications and testimonials, with date of election, to THE EDITOR, The Lodge, Porchester Square, W.]

BELFAST HOSPITAL FOR SICK CHILDREN.—Miss Stopford, late Sister in the Army Nursing Staff, has been appointed Lady Superintendent of the above hospital from a very large number of candidates. Miss Stopford was trained at the Meath Hospital, Dublin, and then went as Lady Superintendent of County Carlow Infirmary, which position she resigned to join the Army Nursing Service. She was Sister at the Royal Victoria Hospital, Netley, for one year, and was then drafted to the Royal Military Infirmary, Dublin, where she remained for upwards of two years, and resigned on her appointment at Belfast on May 25th, 1891.

COPENHAGEN.—Sister Sophie Zahrtmann has been appointed Mother Superior of the Drakonissen House.

LONDON HOSPITAL.—Miss Caroline Linnell has been appointed Sister Mary; Miss M. Venables Hirst has been appointed Sister Cotton; both trained at the London.

TWICKENHAM.—Miss Ada Fuller has been appointed nurse at St. John's Hospital in the place of Nurse Eva Elliott, who resigns after five years' good work.

### Wants and Workers.

[Under this heading, we propose to try whether we can be useful to our readers in making the wants of some known to others who are willing to do what work they can to aid the great cause of curing and cheering the sick. Wants can only be inserted from those who are connected with some institution or association, or who are willing to have their full name and address printed.]

*J. M.* would forward THE HOSPITAL weekly to any charitable institution needing it.

*St. Luke's Home.*—Sister Frances acknowledges from Mr. Postlethwaite and Mr. Harris one guinea each; from Mrs. Shaw £1; from Miss Johnston and Mrs. Turner 5s. each; from Miss Greaves a cloak, a dress, eighteen yards of cretonne and some frames and photographs; from Miss Hill a paper knife; from Mrs. Goslett scarlet flannel dressing jackets; from Mrs. Jack an electric battery; from Miss Johnston a feeding cup; from Mrs. Cole a silver crucifix, "In Memoriam"; and several letters promising help. Further contributions in money, or in any articles useful for hospital work will be gladly received up to August 4th by Sister Frances, Corgrig, High Park, Ryde.

LADY SUPERINTENDENT, King's Lynn Hospital, begs to thank "Anonymous," Newchurch, for two pretty scrap books; also "A. G. F." for his kind promise of pictures. Will not others help? Red jackets, presents for Christmas, &c., would be thankfully received.

### Notes and Queries.

TO CORRESPONDENTS.—1. Questions or answers may be written on post-cards. 2. Advertisements in disguise are inadmissible. 3. In answering a query please quote the number. 4. A private answer can only be sent in urgent cases, and then a stamped addressed envelope must be enclosed. 5. Every communication must be accompanied by the writer's full name and address, not necessarily for publication. 6. Correspondents are requested to help their fellow nurses by answering such queries as they can.

### Queries.

(26) Wanted, the addresses of any nursing institutions in Florence or Switzerland.—*S.D.*

(27) Can anyone suggest an employment for a deaf lady who has a knowledge of shorthand and bookkeeping?—*Nurse T.*

(28) Will any nurse or doctor kindly tell me of a really happy permanent home (free) for a homeless incurable little cripple boy of nine years near London.—*E.J.B.*

### Answers.

(23).—Write to the Lady Superintendent 3, Crosby Road East, Southport.

(25).—It is not necessary to refill water beds unless they get quite cold J. N., had them in use ten months without refilling.—*M Lovesy.*

*Maude K., M.E.S. and others.*—Dr. Duncan Greenless, Grahamstown Asylum, Cape Colony.

*Sister D.*—Sister Mary, of Guy's, has an institution at Biarritz; we have put a query to see if we can get you other addresses.

*Mable.*—We never prescribe.

*Nurse B.*—Apply to Colonel John Robertson, 27, Inverness Terrace, Hyde Park, W., or to the Under Secretary of State for India, Whitehall. You will have to go when you are sent.

(24).—Comfortable lodgings for nurses can be had at Mr. Claxon's, 53, Crown Road, Great Yarmouth.—*Nurse Halliday.*

### An Urgent Case.

*(Concluded from page c.)*

Twelve o'clock struck, and he awoke from dreamland.

A ring at the bell, and a voice, shrill and clear, calling to him through the speaking-trumpet. He slipped into his clothes, with the dexterity acquired by frequent practice, and opened the front door briskly. There stood before him a little boy, poorly clad, with a mop of fair hair and troubled blue eyes.

Those of the doctor softened, and his voice, a trifle sharp and imperative, grew suddenly gentle, as he put his question encouragingly.

"What is it, my little man?"

"Father has been and broken his leg, sir."

The child's tones were solemn, and a tear glistened upon his long lashes.

"Dear me! dear me!" quoth Dr. Trent, "that is a bad job, and no mistake! How did it happen?"

"He was a-comin' home from the 'Pig and Whistle,' and he stumbled over somethink or other, and they just had to carry him home, and mother ——"

"Well, what about mother?"

"Mother, she said as it was all along of his staying out so late."

The boy glanced over his shoulder to see if his companion was following, and the latter, nodding reassuringly, quickened his pace to keep up with him.

"Is your father in much pain?" he asked.

"*Rather*, he is a-swearin' orful."

"No sense in that; less than no sense," commented the doctor, fretfully.

The way was long and dreary, the rain fell fast, the deserted streets looked dull and cheerless, yet the two pushed on bravely. The doctor, finding it impossible to hold up an umbrella in such a wind, resigned himself to a wetting, and got what he expected.

At last his guide halted before a low, thatched dwelling-place, poor and mean of aspect, and the two looked into one another's faces.

"Here we are; and a good job, too," said the youngster.

His companion, realising for the first time how very young he was, not over seven, in spite of his old-fashioned ways, gazed upon him pityingly.

"You are a brave little chap, to have come so far to fetch me at this hour of the night," he said.

The blue eyes flashed at such unexpected praise, the diminutive figure was drawn up to its full height, and the little chap dived his small hands into his capacious pockets.

"Well, you see," replied he, "if a thing is broke, why the best way is to see and get it mended."

"Right you are, my lad! A true philosopher. Let us go inside."

The child rapped with his knuckles upon the battered doorway, and the two were immediately admitted by a buxom woman, who fell back in extreme astonishment.

"Well I never!" cried she, "if it ain't Dr. Trent!"

"Yes, I've been and gone and fetched him," said the lad, triumphantly. "See here, father, here's the doctor!"

A man, seated by the hearth, raised his eyes curiously. He was a gaunt man, a trifle red in the face, probably from stooping, for his occupation seemed to require close scrutiny.

"I'd get up and welcome you, sir," said he, a bit sheepishly, "but I can't, seeing as I've had the misfortune to break my leg. I'm a-trying to mend it now, for I must be up and stirring early to-morrow morning, but I ain't much of carpenter, as the saying is."

"Nor I, either," put in Dr. Trent, bursting into a fit of laughter, as he took in the situation, "but two heads are better than one, and I am willing to lend a hand, though I doubt whether I should have been quite as ready to turn out on a night like this if I had known your broken leg was a wooden one."

"Was it my boy Jerry as fetched you?" queried the man.

"To be sure," assented Dr. Trent, laughing, and in spite of their respect for "doctor," husband and wife joined in his merriment.

Not so Jerry! He glanced from one to the other with anxious, wide-open eyes, but saw no joke.

Neither did Miss Mary Anne when she heard of the transaction.

"It is all of a piece," grumbled she, "nothing but foolishness from first to last. What is the use of a man working himself to skin and bone for no pay?"

Dr. Trent did not answer, neither did his poor patients; but the folks who hold their peace are often those who know the most.

### Death in Our Ranks.

We regret to announce the death of Nurse Dickins, of the Cambridge Institution, who died while on a visit at Lamport after a few days' illness at the age of 29. She was thoughtful, kind, attentive to her patients, and the loss is keenly felt by all who knew her.

From Germany we hear of the death of Frau Aders, known as the German Florence Nightingale. She served through the Franco-German War and won the First Class of the Louise Order and the Service Cross for Women.

### Amusements and Relaxation.

**SPECIAL NOTICE TO CORRESPONDENTS.**

**Third Quarterly Word Competition commenced July 4th, 1891, ends September 26th, 1891.**

Competitors can enter for *all* quarterly competitions, but no competitor can take more than *one first prize* or *two* prizes of any kind during the year.

Proper names, abbreviations, foreign words, words of less than four letters, and repetitions are barred; plurals, and past and present participles of verbs, are allowed. Nuttall's *Standard* dictionary only to be used.

N.B.—Word dissections must be sent in WEEKLY not later than the first post on Thursday to the *Prize Editor*, 140, Strand, W.C., arranged alphabetically, with correct total affixed.

The word for dissection for this, the FIFTH week of the quarter, being

"SKETCHES."

| Names. | July 23rd. | Totals. | Names. | July 23rd. | Totals. |
|---|---|---|---|---|---|
| Paignton | 17 | 95 | Christie | — | 44 |
| Psyche | 18 | 98 | Dulcamara | 17 | 90 |
| Hope | — | 47 | Nurse J. S. | 16 | 93 |
| Lightowlers | 17 | 97 | Qu'appelle | 17 | 93 |
| Wizard | 16 | 91 | E. M. S. | — | — |
| Wyameris | — | 46 | Jenny Wren | 12 | 78 |
| Dove | 16 | 62 | Carpe-diem | — | — |
| Punch | 17 | 91 | Grannie | — | 36 |
| Ivanhoe | 17 | 94 | Nurse G. P. | 11 | 69 |
| Tinie | 16 | 93 | Goodnight | 11 | 52 |
| Agamemnon | 17 | 95 | Gamp | 11 | 31 |
| Nurse Ellen | 15 | 86 | Charity | — | — |

N.B.—Each paper must be signed by the author with his or her real name and address. A *nom de plume* may be added if the writer does not desire to be referred to by us by his real name. In the case of all prize-winners, however, the real name and address will be published.

All letters referring to this page which do not arrive at 140, Strand, London, W.C., by the *first post on Thursdays*, and are not addressed PRIZE EDITOR, will in future be disqualified and disregarded.

## Figures and Tables

**Figure f1:**